# Clinical and dosimetric outcomes of image-guided, dose-painted radiotherapy in muscle invasive bladder cancer

**DOI:** 10.1186/s13014-023-02338-w

**Published:** 2023-09-20

**Authors:** Inmaculada Navarro-Domenech, Shinthujah Arulanantham, Zhihui Amy Liu, Michael Tjong, Vickie Kong, Victor Malkov, Tony Tadic, Neil Fleshner, Girish Kulkarni, Alexandre R Zlotta, Charles Catton, Alejandro Berlin, Srikala Sridhar, Di (Maria) Jiang, Peter Chung, Srinivas Raman

**Affiliations:** 1 Department of Radiation Oncology, Radiation Medicine Program, Princess Margaret Cancer Centre, 610 University Avenue, Toronto, ON M5G 2M9 Canada; 2 Department of Biostatistics, Princess Margaret Cancer Centre, Toronto, Canada; 3 Department of Surgical Oncology, Princess Margaret Cancer Centre, Toronto, Canada; 4 Department of Medical Oncology and Hematology, Princess Margaret Cancer Centre, Toronto, Canada

**Keywords:** Bladder cancer, Radiotherapy, Dose-painted, Lipiodol, Tri-modal therapy

## Abstract

**Purpose/Objective:**

Definitive radiotherapy (RT) is an alternative to radical cystectomy for select patients with muscle invasive bladder cancer (MIBC); however, there is limited data on dose-painted RT approaches. We report the clinical and dosimetric outcomes of a cohort of MIBC patients treated with dose-painted RT.

**Material/Methods:**

This was a single institution retrospective study of cT2-4N0M0 MIBC patients treated with external beam radiotherapy (EBRT) to the bladder, and sequential or concomitant boost to the tumor bed. The target delineation was guided by either intravesical injection of Lipiodol or through fusion of the pre-treatment imaging. The majority were treated with daily image-guidance. Kaplan-Meier was used to characterize overall survival (OS) and progression-free survival (PFS). Cumulative incidence function (CIF) was used to estimate local (intravesical) recurrence (LR), regional recurrence (RR) and distant metastasis (DM). Univariable and multivariable cause-specific hazard model was used to assess factors associated with LR and OS.

**Results:**

117 patients were analyzed. The median age was 73 years (range 43, 95). The median EQD2 to the boost volume was 66 Gy (range 52.1, 70). Lipiodol injection was used in 64 patients (55%), all treated with IMRT/VMAT. 95 (81%) received concurrent chemotherapy, of whom, 44 (38%) received neoadjuvant chemotherapy. The median follow-up was 37 months (IQR 16.2, 83.3). At 5-year, OS and PFS were 79% (95% CI 70.5–89.2) and 46% (95% CI 36.5–57.5). Forty-five patients had bladder relapse, of which 30 patients (67%) were at site of the tumor bed. Nine patients underwent salvage-cystectomy. Late high-grade (G3-G4) genitourinary and gastrointestinal toxicity were 3% and 1%.

**Conclusion:**

Partial boost RT in MIBC is associated with good local disease control and high rates of cystectomy free survival. We observed a pattern of predominantly LR in the tumor bed, supporting the use of a dose-painted approach/de-escalation strategy to the uninvolved bladder. Prospective trials are required to compare oncological and toxicity outcomes between dose-painted and homogeneous bladder RT techniques.

## Introduction

Bladder cancer is one of the most common cancers worldwide, with over 550,000 cases diagnosed annually [1]. Approximately one-third of these are muscle-invasive bladder cancers (MIBCs), which carry a poor prognosis and require aggressive multi-modal management [2, 3]. Traditionally, curative treatment involves perioperative chemotherapy and radical cystectomy (RC). Not all patients are candidates for this approach however, potentially due to age, comorbidities and/or preference to avoid cystectomy [4–7]. An alternative approach for bladder preservation is the combination of maximal trans-urethral resection of the bladder tumour (TURBT) followed by definitive radiation treatment (RT) [8, 9]. Although no prospective randomized trials have been successfully completed comparing survival outcomes after RC and RT in MIBC, there are other high quality studies to support oncological equivalence between the two treatments in appropriately selected patients [8, 10–12].

Although most of the RT protocols published in the literature deliver a uniform RT dose to the whole bladder [13, 14], modern advances in radiotherapy techniques can facilitate dose-painted approaches [15, 16]. Uniform whole bladder RT is supported by two randomized studies, which compared this approach with partial / dose-painted radiotherapy [13, 14]. These studies did not show toxicity differences, and additionally the non-inferiority in loco-regional control with partial / dose-painted RT could not be concluded. However these trials predated the use of image-guided and Intensity modulated radiotherapy. With the use of image-guided radiotherapy (IGRT) and intensity modulated radiotherapy (IMRT), as well as adaptive RT and fiducial markers to enable precision delivery, we hypothesize that dose-painted RT may be associated with comparable oncological outcomes to other treatments with a favourable toxicity profile.

While several studies have reported on the oncological outcomes of whole bladder RT protocols, there is limited data on the outcomes of dose-painted approaches; and in particular on the patterns of recurrences in these patients [17–19]. Information about patterns of recurrence with dose-painted RT may guide further optimization of treatment techniques and follow-up protocols; and ultimately improve their uptake in appropriately selected patients. In this study, we report on the clinical and dosimetric outcomes of a large cohort of MIBC patients treated with dose-painted RT.

## Methods

### Study design and patient characteristics

After obtaining institutional research ethics board (REB) approval, we performed a retrospective single institution study of selected patients with a diagnosis of cT2-T4 MIBC, treated with definitive radiotherapy between April 2003 and November 2020. In this study, we only included patients who received dose-painted radiotherapy, and patients with uniform prescription doses to the bladder were excluded.

All eligible patients were assessed in a multi-disciplinary bladder clinic, and as part of pre-treatment evaluation, they underwent a history and physical examination, pathology review and systematic staging with CT thorax-abdomen-pelvis. Bone scan and/or pelvis MRI were performed at physician discretion. General patient selection criteria for radical RT, with or without radiosensitizing chemotherapy (single-agent cisplatin or gemcitabine), at our institution include previous maximal TURBT, transitional or squamous cell carcinoma histology, good bladder function, tumor size ≤ 7 cm, absence of severe hydronephrosis, absence of multifocal carcinoma-in-situ (CIS) and no contraindications to pelvic radiotherapy. The same general criteria applied to patients treated with a dose-painted approach, with the additional requirement of being able to delineate the tumor bed with Lipiodol and / or cross sectional imaging. For follow up, the patients underwent cystoscopy surveillance 6–8 weeks post-radiotherapy and then every 3 months for the first 2 years; every 6 months for the 3rd to 5th year and then annually. Imaging follow was performed with CT thorax-abdomen-pelvis at 3 months, 6 months, 12 months, 18 months, 24 months and then annually.

### Radiation treatment

All patients underwent CT simulation with comfortably full bladder and empty rectum [20]. Clinical target volume (CTV) for the low dose level included the whole bladder (CTVbladder). The CTV for the boost region (CTVboost) was delineated manually by a radiation oncologist, and majority of patients underwent Lipiodol contrast injection to guide this process. The Lipiodol contrast (0.5-1 ml) was injected by the urologist through a cystoscopy procedure at 12, 3, 6 and 9’0 clock at roughly 1 cm away from the TURBT scar prior to CT simulation. Treatment of the pelvic lymph nodes was at the investigator’s discretion. If performed, the areas included the internal/external iliac, distal common iliac, obturator, and presacral regions [20].

Different dose prescriptions and protocols were utilized during the time period of the analyzed data, and are detailed in the results section. The most common boost doses for conventionally fractionated (CF) and hypofractionated (HF) protocols were 66 Gy in 33 fractions and 55 Gy in 20 fractions, respectively. The most common low dose levels for CF and HF protocols were 60 Gy in 30 fractions and 50 Gy in 20 fractions, respectively. Initially, patients were treated with 4-field technique until 2010 when, parallel to the advancement of techniques within the institution, conformal treatments with 3D conformal radiotherapy (3DCRT), IMRT or volumetric modulated arc therapy (VMAT) were used. In 2006, the use of daily cone beam CT (CBCT) for image guidance was introduced on clinical trial (NCT00913536), and became routine for all patients since 2008. Early plans included a whole bladder uniform 2 cm planning target volume (PTV) margin (PTVbladder), and the PTV for the boost volume (PTVboost) was a 1.5- 2 cm expansion around the CTV. In 2019, an adaptive protocol and reduction of margins were implemented. An internal target volume (ITV) for the bladder and the boost region (ITVbladder and ITVboost, respectively), was generated using the CBCT from fractions 1–4. Both the bladder and the boost volumes were expanded by 5 mm on the ITV to generate the PTV.

### Statistical analysis

Summary statistics were used to describe patient, disease and treatment characteristics. For continuous variables, mean, standard deviation (SD), median, interquartile range (IQR) and range were calculated. For categorical variables, frequency and percentage were presented. Overall survival (OS) and progression-free survival (PFS) were estimated using the Kaplan-Meier (KM) method, and log-rank test was used for group comparisons. Cumulative incidence function (CIF) was used to estimate local recurrence (LR), regional recurrence (RR), distant metastasis (DM) with death as a competing risk; Gray’s test was used for group comparisons. Cancer-specific mortality (CSM) was estimated using CIF with death due to other causes as competing risk. Univariable and multivariable cause-specific hazard model was used to assess factors associated with local recurrence. Due to the retrospective nature of this analysis and transition between treatment planning systems during the study period, there is some dosimetry data missing. As such, the dosimetry was analyzed with the available data. All tests were two-sided and a p-value ≤ 0.05 was considered statistically significant. Statistical analyses were conducted in R v4.0.2 [21].

## Results

A total of 117 patients with MIBC clinical stage T2-4N0M0 were analyzed. All of them underwent an initial TURBT demonstrating transitional cell carcinoma in 114 patients (97.4%) and squamous cell carcinoma in 3 patients (2.6%). The median age was 73 years (range 43, 95). Patient demographics are detailed in Table 1.

**Table 1:** Demographic characteristics

Covariate		Total patients (n = 117)
**Sex**	Female	38 (32%)
	Male	79 (68%)
**Smoking history**	No	37 (32%)
	Current	18 (15%)
	Ex-smoker*	53 (53%)
**ECOG**	0	62 (53%)
	1	38 (32%)
	2	12 (10%)
	3	5 (4%)
**Clinical stage**	T2	96 (82%)
	T3	14 (12%)
	T4	7 (6%)
**Histology grade**	G2	4 (3%)
	G3	113 (97%)
Concurrent CIS**	Yes	31 (26%)
	No	86 (74%)
**Hydronephrosis**	Yes	19 (16%)
	No	98 (84%)
**Histology**	TCC	114 (97%)
	Other	3 (3)
**Focality**	Unifocal	85 (73%)
	Multifocal	32 (27%)
**Tumor size**	Median (Q1,Q3)	3 cm (2.0, 3.6)
	> 5 cm	9 (8%)

*Quit at least more than 12 months before diagnosis. ** Carcinoma in situ (CIS)

### Clinical outcomes

**Fig. 1:**
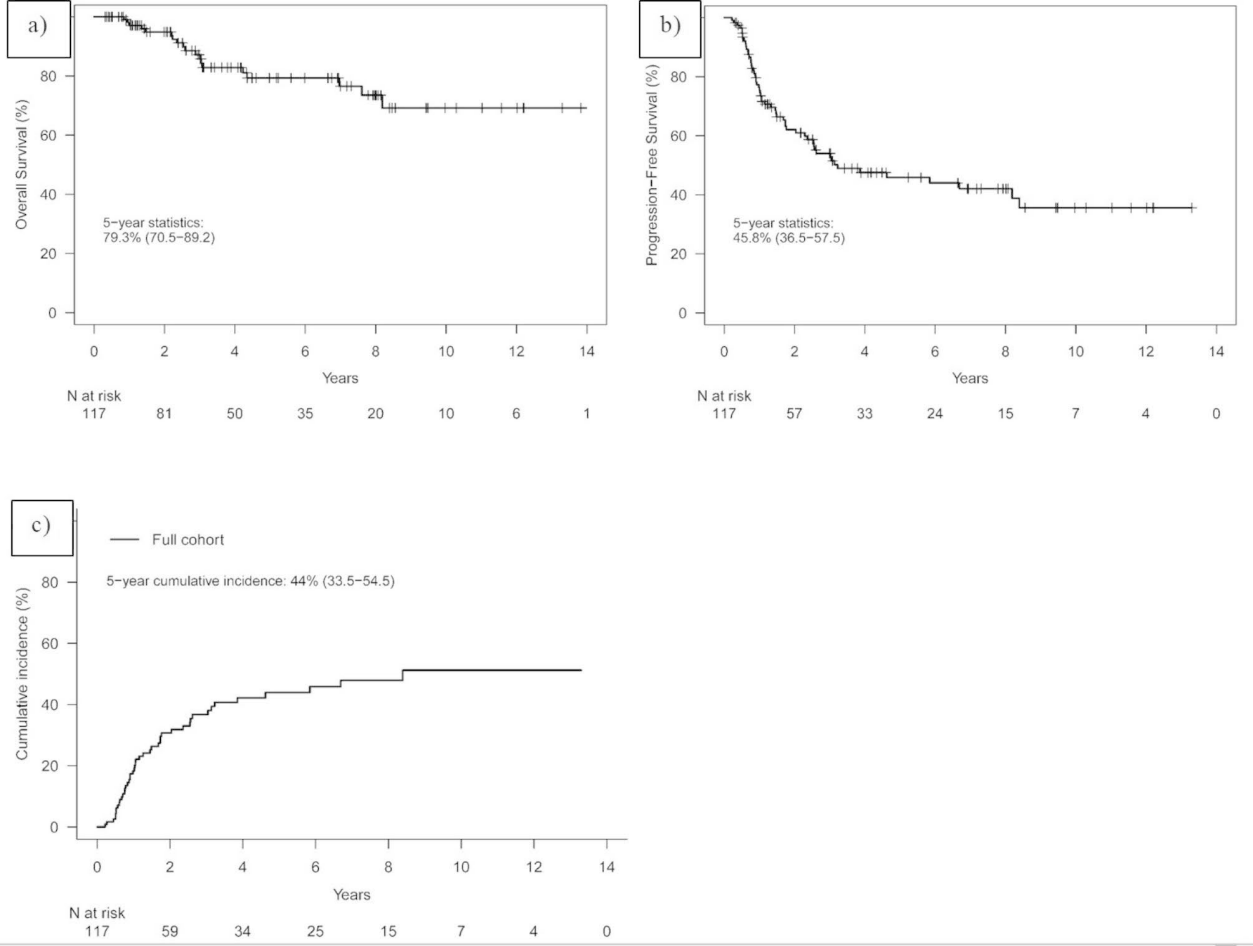
Clinical outcomes: (a) Kaplan-Meier curve demonstrating Overall Survival (OS); (b) Kaplan-Meier curve demonstrating Progression Free Survival (PFS); (c) Kaplan-Meier curve demonstrating Local Recurrence (LR).

The median follow up of the cohort was 36.6 (IQR 16.2, 83.3) months. Overall survival (OS) and progression free-survival (PFS) at 5 years were 79.3% (95% CI 70.5–89.2) and 45.8% (95% CI 36.5–57.5) respectively, as illustrated on the KM curves in Fig. 1. Cancer-specific death occurred in 7 (6.0%) patients. A total of 45 (38%) patients from the entire cohort developed local recurrence and the cumulative incidence (CIF) at 5 years was 44% (95% CI 33.5–54.5), as shown in Fig. 1. Nineteen (42.2%) of them, developed a muscle-invasive recurrence. Thirty of the local recurrences (66.7%) were in the area of the original tumor / scar after TURBT. Nine (7.7%) patients from the entire cohort underwent salvage cystectomy and the 5-year cystectomy free survival in the entire cohort was 89.8%. CIF of regional recurrence at 5 years was 9.7% (95% CI 3.5–15.9) and distant metastases occurred in 22 patients (19%) with a CIF at 5 years of 22.6% (95% CI 14-31.1). In the overall cohort, palliative systemic therapy was utilized in 5 (11%) of the patients with recurrent disease, and 3 (7%) received palliative radiation treatment to metastatic disease.

**Fig. 2:**
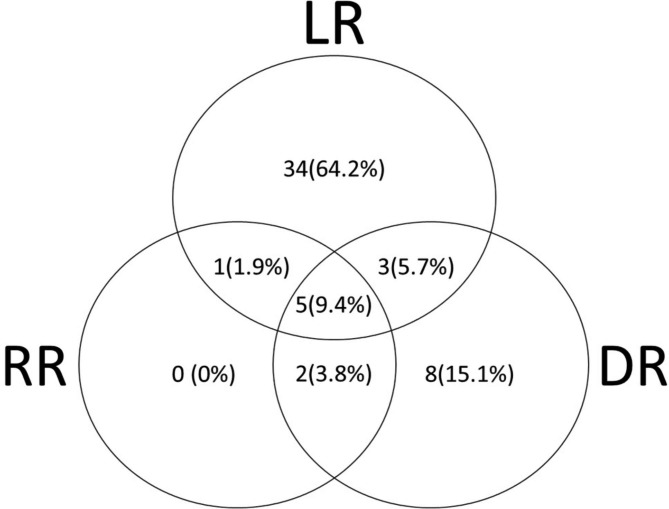
Patterns of local, regional, and distant recurrences at first relapse. Total local recurrences = 43 of 53 (81.1%), total regional recurrences = 8 of 53 (15.1%), and total distant recurrences = 18 of 53 (34.0%). Abbreviations: DR = distant recurrence; LR = local recurrence; RR = regional recurrence

At the time of first recurrence, from the 53 patients that developed any kind of recurrence, 34 patients (64%) had isolated-bladder recurrence. Of these, 12 patients (35.5%) developed invasive local recurrence, 12 (35.5%) had only CIS and 10 (29%) had non-invasive tumor. 31 (91%) patients underwent local salvage treatment and the initial local treatment was: cystectomy [6], endoscopic resection [11] and endoscopic resection + BCG [14]. For the nine patients who underwent cystectomy; six of them were soon after diagnosing recurrent disease, and three of them were delayed after recurrence following TURBT +/- BCG treatments. The patterns of first recurrence are illustrated in Fig. 2.

A univariate and multivariable analysis was performed to identify predictors of local failure. None of patient characteristics (age, sex, smoking and ECOG), tumor characteristics (concurrent CIS, tumor size, hydronephrosis, clinical stage), nor treatment factors (lipiodol use neoadjuvant chemotherapy and concurrent chemotherapy) were statistically significant.

### Dosimetry

**Fig. 3:**
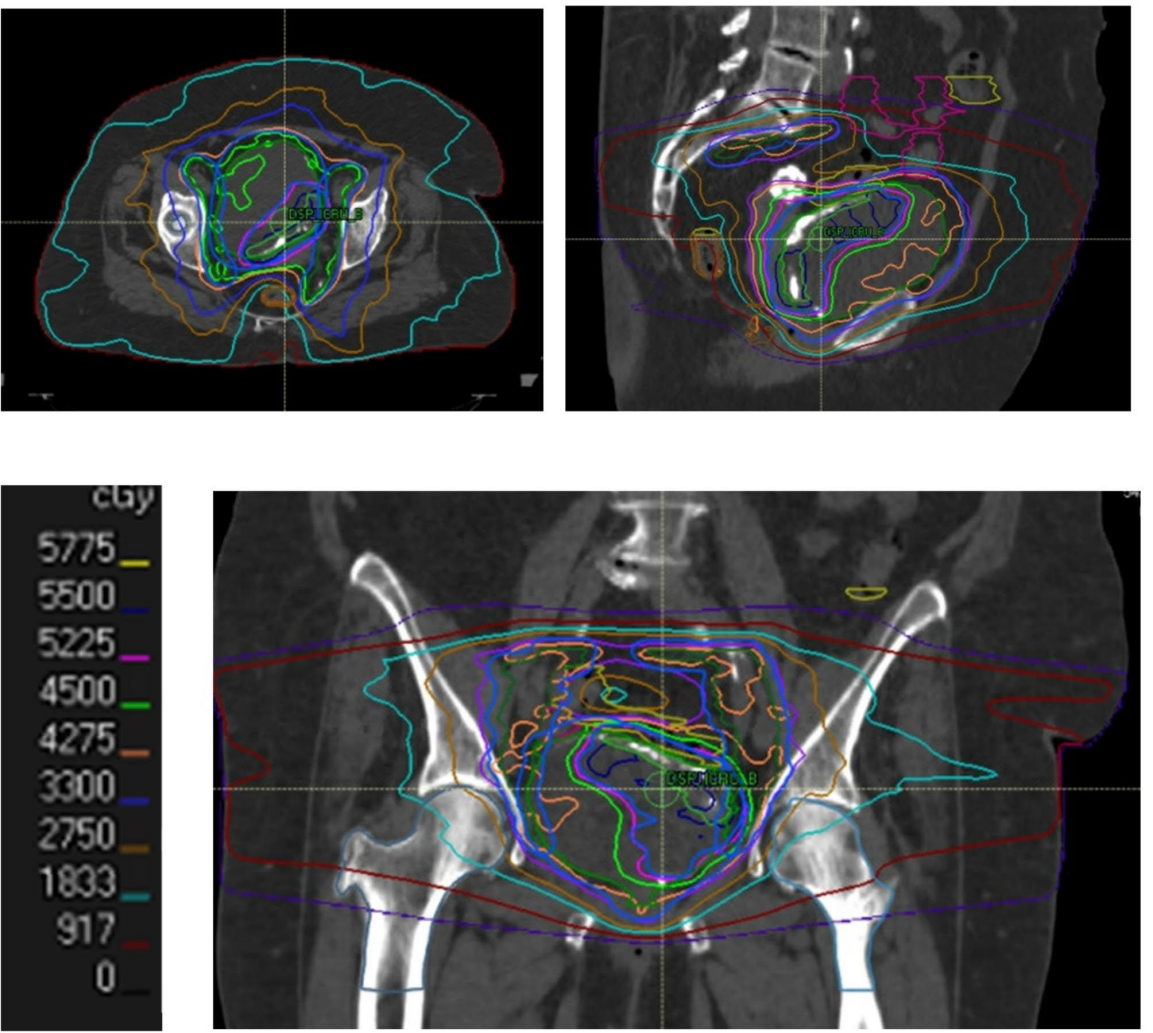
Axial, sagittal and coronal view of a radiation treatment plan to the bladder and elective pelvic lymph nodes chain

All the patients completed external radiation treatment (EBRT) to the bladder (Fig. 3), with a median boost dose of 66 Gy EQD2 (range 52.1, 70). In 107 (91%) of cases, the elective pelvic lymph node chains were treated. As detailed in the methods section, there was a transition from 4-field/3D-CRT to IMRT/VMAT techniques (29 (25%) vs. 88 (75%) patients in each group, respectively), and to CBCT image guidance (103 (88%) patients). Pre-treatment Lipiodol contrast instillation was performed in 64 (55%) patients. Ninety five patients (81.2%) received concurrent chemotherapy along with the radiation, of whom 44 (37.6%) received neoadjuvant chemotherapy as well. No patients received adjuvant chemotherapy. Twenty one (18%) did not receive any chemotherapy due to age, renal failure or other comorbidities. Treatment characteristics are summarized in Table 2.

**Table 2:** Treatment characteristics

Covariate		Full sample (n = 117)
**Chemotherapy**	Concurrent alone	51 (43.6%)
	Neoadjuvant + concurrent	44 (37.6%)
	Neoadjuvant alone	1 (0.9%)
	No chemotherapy	21 (17.9%)
**Pelvic radiotherapy**	Yes	107 (91%)
	No	10 (9%)
**Lipiodol**	Yes	64 (55%)
	No	53 (45%)
**Technique**	3D-CRT	2 (2%)
	4-Field	27 (23%)
	IMRT	76 (65%)
	VMAT	12 (10%)
**CBCT**	Yes	103 (88%)
	No	14 (12%)
**Plan**	2-phase	87 (74%)
	3-phase	26 (22%)
	SIB	4 (3%)

Due to the transition to different planning system (Pinnacle and Raystation), volume and dosimetry data were available in 109 and 70 patients. Dosimetry characteristics in the overall cohort and in the Lipiodol vs. non-Lipiodol groups are summarized in Table 3. In the Lipiodol group, the mean percentage of the whole bladder volume that received the 95% of the total dose was lower compared to the non-Lipiodol group [51.5% vs. 72.6% (p 0.004)]. All the 64 patients who underwent lipiodol instillation were treated with IMRT or VMAT vs. 24 (45%) in the non-Lipiodol group. And 100% of the Lipiodol patients underwent CBCT verification, compared to 39 (74%) in the non-lipiodol group.

**Table 3:** Dosimetry characteristics

Covariate	Overall (n = 117)	Lipiodol (n = 64)	No lipiodol (n = 53)	p-value
**Mean boost clinical target volume (cm3) (sd)** [Missing]	46.0 cc (43.4) [8]	36.8 cc (28.0) [1]	58.6 cc (56.2) [7]	**0.02**
**Mean bladder volume (cm3) (sd)** [Missing]	257.5 cc (126.2) [9]	268.7 cc (113.4) (n = 63) [1]	241.8 cc (142.0) (n = 45) [8]	0.17
**Mean percentage of bladder volume receiving 95% of boost dose (sd)** [Missing]	58.4% (28.3) [47]	51.5% (25.8) [17]	72.6% (28.4) [30]	**0.004**

### Toxicity

The incidence of late radiation high-grade (G3-G4) toxicity was analyzed retrospectively. Genitourinary (GU) toxicity was present in four patients (3%), and gastrointestinal toxicity (GI) in one patient (1%). None of the patients stopped the radiation due to toxicity. Four patients experienced Grade 3 + GU toxicity, with the most common toxicity being symptomatic urinary tract obstruction altering organ function and severe urinary incontinence, requiring operative intervention or permanent urinary catheterization. GI toxicity was rare and the observed case was chronic diarrhea with increase of > = 7 stools per day over baseline, that required hospitalization.

## Discussion

In this study, we report on clinical outcomes and dosimetry parameters in a large cohort of patients who underwent dose-painted radiotherapy at our institution. Overall, the patients had very good oncological outcomes with 5 year OS of 79% and PFS of 46%. Accounting for differences in patient characteristics, these outcomes compare favourably to other reported series of bladder sparing therapy [16], [20].

The most common and first site of recurrence after definitive radiotherapy is local, underscoring the need for close cystoscopy follow-up and consideration of salvage local treatment. Furthermore, the majority of local failures occurred at the site of original tumor involvement. These results are consistent with other studies also confirming predominantly, in-field local patterns of failure. For example Abdel-Aty et al. utilized image registration to map patterns of local failure, and found that that the majority of local recurrences occurred at or within close proximity of the original tumour [22].

The use of neoadjuvant chemotherapy (NAC) may be associated with improved survival in patients undergoing surgery or radiation therapy [23, 24]. One institutional series of patients undergoing neoadjuvant chemotherapy, followed by definitive chemoradiotherapy [8], published good tolerability of treatment, with most patients completing a full course of NAC (95%) and all patients completing their planned course of radiotherapy. As per institutional policy, most eligible patients also received concurrent chemotherapy, which is now definitively the accepted standard of care to optimize locoregional control [25]. In our series, 45 patients received NAC, and it was associated with a lower rate of local recurrence in the univariate and multivariate analysis.

Most protocols currently utilize a uniform dose approach. Previous randomized trials comparing partial bladder radiotherapy to whole bladder radiotherapy did not show a difference in local control or toxicity outcomes [14, 26]. However these trials did not utilize modern radiotherapy techniques such as IMRT or IGRT. In our cohort, the majority of patients were treated with IMRT/VMAT (75%) and underwent daily CBCT (88%). At our institution, we have also adopted the use of lipiodol fiducial markers in eligible patients since 2009. Several groups, including our own, have demonstrated the feasibility, safety and potential gains of using Lipiodol for target delineation and as a fiducial marker for image guidance [20]. In our study, the use of Lipiodol was associated with smaller CTV’s and improved uninvolved bladder sparing compared to those without Lipiodol. The CTV reduction from Lipiodol use was likely secondary to reduce uncertainly in target delineation. The improvement in uninvolved bladder sparing is multifactorial, related to CTV reduction, planning margin reduction and increased use of IMRT and IGRT in the Lipiodol cohort. In addition to target delineation and planning technique, the choice of planning target margins can impact the delivered dose. Previous study has also previously demonstrated that use of patient-specific PTV along with cone beam CT facilitated large reductions in the irradiated normal tissue volume without compromising target coverage [27]. We have adopted the use of patient-specific PTV margins since 2015, and our most recent bladder adaptive protocol combines the CTV’s from the first four CBCT, to generate an ITV and then expanded of 5 mm in all directions to create the patient-specific PTV for the remainder of treatment. We demonstrated low rate of late high grade toxicity in this cohort, which may be related to the decrease in CTV.

The majority of patients treated in our study were treated with conventional fractionation. During the COVID-19 pandemic in 2020, the default prescription for most patients changed to 55 Gy in 20 fractions. Recently published individual patient data meta-analysis of the BC2001 and BCON trials suggested that hypofractionated radiotherapy protocol of 55 Gy in 20 fractions was superior in regards to invasive locoregional control compared to a conventionally fractioned radiotherapy protocol of 64 Gy in 32 fractions [28]. This association could not be tested in our current dataset, given limited numbers of patients treated with hypofractionation and short follow up on these patients. Another association that could not to be ascertained is the relationship between elective nodal RT and outcomes, given that 91% of patients received pelvic RT. The impact on disease control and gastrointestinal toxicity are unclear, and the low rates of late grade 3 + GI toxicity (1%) have influenced institutional practice in continuing to offer elective pelvic RT for most patients.

With regards to future directions, investigators from UK have designed an international phase II trial to compare standard whole bladder radiotherapy vs. dose adaptive tumour-focused Radiotherapy vs. standard dose-escalated adaptive tumour-focused radiotherapy [13]. The rationale for dose escalation to the tumor bed in this trial is supported by the predominantly local patterns of failures observed in our study. To further improve the therapeutic ratio of bladder radiotherapy, our team has designed a prospective adaptive radiotherapy trial (NCT03909893) which utilizes cone beam CT or on treatment MRI to adapt the RT plan. Various systemic therapy strategies are also currently under investigation to improve oncological outcomes. Specifically, the use of immunotherapy has shown to have significant benefit in high risk patients undergoing radical cystectomy [29]. Several phase II trials have demonstrated a promising role for integrating checkpoint inhibitors into trimodality treatment for MIBC, and results are awaited from randomized trials (NCT03775265, NCT03768570, NCT04241185).

Ongoing improvements and innovations in radiotherapy techniques / technology and systemic therapy may further improve the therapeutic ratio of bladder radiotherapy. The utilization of external beam radiotherapy as, an alternative to cystectomy, is increasing in several jurisdictions, and is supported by accruing evidence of equivalent oncological outcomes between both treatments in appropriately selected patients, while recognizing that both treatments can adversely quality of life (QOL) through different ways [30, 31]. Early studies evaluating QOL after bladder sparing radiotherapy consistent demonstrate a small but measureable detriment in physical, emotional and social domains after treatment. More recent studies using contemporary radiotherapy techniques shows that QOL returns to baseline by 6 months in the majority of patients [32]. Further research is required to determine the clinical advantages of dose-painted radiotherapy approaches and adaptive protocols in the era of contemporary radiotherapy techniques and evolving systemic therapies.

## Conclusion

The favourable oncological and toxicity outcomes in this study, along patterns of recurrence analysis confirming predominantly local failure pattern support the use of a dose-painted approach / de-escalation strategy to the uninvolved bladder. Further investigation is required to test the comparative efficacy of dose-painted and homogenous bladder dosing approaches in the contemporary era of IMRT and IGRT utilization.

## Data Availability

Research data are not publicly available, but interested parties are welcome to contact the corresponding author to discuss the possibility of a mutual data-use agreement.

## Abbreviations

3DCRT: 3D conformal radiotherapy
BCG: Bacillus Calmette-Guerin
CSM: Cancer-specific mortality
CIS: Carcinoma-in-situ
CTV: Clinical target volumen
CBCT: Cone beam CT
CF: Conventionally fractionated
CIF: Cumulative incidence function
DM: Distant metastasis
ECOG: Eastern Cooperative Oncology Group
EBRT: External beam radiotherapy
GI: Gastrointestinal
GU: Genitourinary
HF: Hypofractionated
IGRT: Image-guided radiotherapy
IMRT: Intensity modulated radiotherapy
ITV: Internal target volumen
KM: Kaplan-Meier
LR: Local recurrence
MIBC: Muscle invasive bladder cancer
NAC: Neoadjuvant chemotherapy
OS: Overall survival
PFS: Progression-free survival
QOL: Quality of life
RC: Radical cystectomy
RT: Radiotherapy
RR: Regional recurrence
TURBT: Trans-urethral resection of the bladder tumour
VMAT: Volumetric modulated arc therapy
